# Leg-length discrepancy is associated with low back pain among those who must stand while working

**DOI:** 10.1186/s12891-015-0571-9

**Published:** 2015-05-07

**Authors:** Satu Rannisto, Annaleena Okuloff, Jukka Uitti, Markus Paananen, Pasi-Heikki Rannisto, Antti Malmivaara, Jaro Karppinen

**Affiliations:** Center for Life Course Epidemiology and Systems Medicine, University of Oulu, Oulu, Finland; Medical Research Centre Oulu, University of Oulu and Oulu University Hospital, Oulu, Finland; Finnish Institute of Occupational Heath, Health and Work Ability, Tampere, Finland; Finnish Social Science Data Archive, University of Tampere, Tampere, Finland; Clinic of Occupational Medicine, Tampere University Hospital, Tampere, Finland; School of Health Sciences, University of Tampere, Tampere, Finland; School of Management, University of Tampere, Tampere, Finland; Centre for Health and Social Economics, National Institute for Health and Welfare, Helsinki, Finland; Finnish Institute of Occupational Heath, Health and Work Ability, Oulu, Finland

**Keywords:** Leg length discrepancy, Low back pain, Meat cutters, Standing work

## Abstract

**Background:**

Some studies suggest that leg length discrepancy (LLD) is associated with low back pain (LBP) but many have not found such an association leading to conflicting evidence on the role of LLD in LBP.

**Methods:**

The study population consisted of meat cutters with a standing job and customer service workers with a sedentary job from Atria Suomi Ltd (Nurmo, Finland) who were at least 35 years old and had been working in their jobs for at least 10 years. Leg length of each participant was measured with a laser range meter fixed in a rod, which was holding the scanning head of the ultrasound apparatus. Association of the intensity of LBP (10-cm Visual Analog Scale) with LLD was analysed by linear regression model, while the hurdle model was used in analysing the association of number of days with LBP and days on sick leave during the past year. Associations were adjusted by gender, age, BMI, smoking, depressive feelings and type of work (standing or sedentary job).

**Results:**

The final study population consisted of 114 meat cutters (26 females and 88 males) and 34 customer service workers (30 females and four males). Forty-nine percent of the meat cutters and 44% of the customer service workers had LLD of at least 6 mm, while 16% and 15%, respectively, had LLD of at least 11 mm. In the whole study population, LLD of 6 mm or more was associated with higher intensity of LBP and number of days with LBP. In the stratified analysis, both intensity of LBP and number of days of LBP were associated with LLD among meat cutters but not among customer service workers. The sick leaves during past year were slightly longer among those with LLD 10 mm or more, but the differences were not statistically significant.

**Conclusions:**

LLD, measured with a laser range meter, was associated with intensity of LBP and self-reported days with LBP during the past year among meat cutters engaged in standing work.

**Trial registration:**

ISRCTN11898558 - The role of leg length discrepancy in low back pain.

## Background

Low back pain (LBP) is the most debilitating condition globally, presenting with severe socioeconomic and health-care consequences [1,2]. The etiology of non-specific LBP is not well understood. Leg-length discrepancy (LLD) has been listed as one risk factor of LBP although its role as such has been questioned [3]. The reason for doubt is the contradictory data published as several studies have found a relation between LLD and LBP [4-6] but on the other hand many have not found such an association [7-10].

One explanation for the conflicting evidence on the role of LLD in LBP may be the diversity in measurement methods. Radiographic measurement of LLD has been traditionally regarded as the gold standard but radiation exposure is potentially harmful and therefore radiographic LLD measurements have become less popular. The ultrasound technique has been reported as reliable when compared to radiographic measurement [11]. Recently, we found that a modified ultrasound method was reliable and its agreement with radiographic method was excellent [12].

The aim of the current study was to evaluate the association between LLD and LBP using the novel ultrasound method in an occupational population consisting of meat cutters and customer service workers. We hypothesized that meat cutters, standing mostly in their work, would more likely experience LBP and pain-related consequences in case of LLD.

## Methods

### The study population

The study population consisted of workers in the food industry (Atria Suomi Ltd, Nurmo, Finland). We selected for the study meat cutters with a standing job and customer service workers with a sedentary job. All the workers from those two departments who were at least 35 years old and had been working in their jobs for at least 10 years were invited to the study. The exclusion criteria were rheumatoid arthritis and a history of leg fractures. The study was approved by the Ethics Committee of the Central Hospital of Southern Ostrobothnia (11/2006), and followed the principles of the Declaration of Helsinki. All participants took part on a voluntary basis and signed their informed consent.

### Measurement of leg-length discrepancy

Two physiotherapists from Seinäjoki University of Applied Sciences measured the legs of the participants during July in the summer 2008. They were unaware of the participants’ LBP status. The laser range meter (Hilti, model PD 30, Kaufering, Germany) was fixed in a rod which was holding the scanning head of the ultrasound apparatus (Shimadzu diagnostic ultrasound, model SDU-350A, Sydney, Australia) and could be moved automatically by the linear actuator (HIWIN, model LAM3-4, Taichung, Taiwan). The height of the stem was 1200 mm. The frequency of the scanning head was 3.5 MHz. The scanning head was placed perpendicular to the tissue interface in the hip area. The distance to the floor was measured by the laser measure at the point of the highest rim of the femoral head.

### Demographics and outcome data

Before the two physiotherapists measured the length of legs the participants received a questionnaire containing items on occupational history and lifestyle factors such as smoking habits and depressive feelings. Their body weight (kg) and height (m) were measured. The Body Mass Index (BMI) was calculated as weight (kg) divided by height squared (m^2^).

The participants were categorized as current smokers if they smoked at least 2 days a week. We used patient health questionnaire (PHQ-9) to evaluate the frequency of depressive feelings during the past two weeks using a four-grade categorization: 0) not at all, 1) several days, 2) more than half of the days, 3) nearly every day. It consisted of nine items; “Little interest or pleasure in doing things”, “Feeling down, depressed, or hopeless”, “Trouble falling or staying asleep, or sleeping too much”, “Feeling tired or having little energy”, “Poor appetite or overeating”, “Feeling bad about yourself – or that you are a failure or have left yourself or your family down”, “Trouble concentrating on things, such as reading the newspaper or watching television”, “Moving or speaking so slowly that other people could have noticed. Or the opposite – being so fidgety or restless that you have been moving around a lot more than unusual”, and “Thoughts that you would be better off dead, or of hurting yourself”. Total Score (TS) of 10–14 means moderate depressive feelings, whereas score of 15–19 moderately severe depressive feelings and score of 20–27 severe depressive feelings [13].

The study subjects reported intensity of LBP during the past week and three months using a 10-cm Visual Analogue Scale (VAS), and self-reported number of days with LBP during the past year. The participants have not had prolonged absence from work during these reference periods. They were asked to estimate current work ability compared with the lifetime best on a 11-point numerical rating scale where 0 is worst and 10 best lifetime work ability.

Sickness absence data including diagnoses and duration of the sick leaves due to diagnoses of LBP (M50-54 in ICD 10) during the past five years is collected to the occupational health clinic of Atria Suomi Ltd comprehensively. Sickness absence data was used in the comparison of participants and non-participants.

### Statistical analyses

The gender differences in LLD and height were analysed by the Kruskal-Wallis test and the independent samples t-test, respectively, using Stata (V 13.1). Association of the intensity of LBP with absolute and relative (as a percentage of mean leg length) LLD was analysed by linear regression model using SAS program (V 9.2). Due to its skewed distribution logarithmic transformation was used in the analyses. We used the hurdle model (see below) in analysing the association of number of days with LBP during the past year with absolute and relative LLD. The same model was used for association between the number of days on sick leave during the past year and both LLD variables. Associations were adjusted by gender, age, BMI, smoking, depressive feelings and type of work (standing or sedentary job). The potential influence of type of work on the association between LBP and LLD was explored with interaction terms and conducting the analyses separately for meat cutters and customer service workers. An interaction effect between gender and absolute or relative LLD was also tested in each model.

Count variables are most frequently analysed using Poisson regression but the Hurdle model [14] is a proper method to handle excess of zeros and/or the overdispersion in the data. Hurdle model is a two-component model where a truncated-at-zero count component is employed for positive counts and hurdle component for zero vs. non-zero counts. We used negative binomial model for count component and binomial model with logit link for hurdle component. Hurdle portion of the model estimates the probability of having LBP at least once and count portion estimates the mean number of days of LBP among those who have had LBP during the past year. Parameter estimates of the count and hurdle components are mean ratios and odds ratios respectively. The parameters of hurdle models were estimated using hurdle function in the package pscl in R environment (http://www.r-project.org/).

## Results

The study population consisted of 218 workers; 169 pork meat cutters (31 females and 138 males) and 50 customer service workers (41 females and 9 males). In all, 114 of the 169 meat cutters (68%; 26 females and 88 males) and 34 of the 50 customer service workers (68%; 30 females and four males) participated. Summer vacations were the main reason for refusing to participate in our study. There were no differences between the non-participants and participants with respect to gender, age, working years and sick leave days because of LBP (M50-54 in ICD-10) during the past year and the past five years (data not shown).

The distribution of LLD and characteristics of the total study population and according to the type of work are presented in Table 1. Fifty-five (49%) of the meat cutters and 15 (44%) of the customer service workers had LLD of at least 6 mm, while 16% and 15%, respectively, had LLD of at least 11 mm. LLD of at least 6 mm was more common among males (55%) than females (37%) and the gender difference was statistically significant (p < 0.05). LLD relative to leg length did not differ significantly between genders (p > 0.05).

**Table 1:** **Characteristics (%/mean) of the whole study population and according to type of work**

	**All**			**Customer service workers**			**Meat cutters**		
	**All**	**Female**	**Male**	**All**	**Female**	**Male**	**All**	**Female**	**Male**
**Total number**	148	61	87	34	29	5	114	32	82
**Age (years)**	47	49	46	48	49	40	47	48	46
**Height (m)**	1.73	1.65	1.78	1.68	1.66	1.78	1.74	1.64	1.78
**LLD (mm)**	5.4	4.5	6.0	5.4	5.2	6.6	5.4	3.8	6.0
**LLD > 5 mm**	48	37	55	44	45	40	49	29	56
**LLD > 10 mm**	16	11	18	15	10	40	16	13	17
**Relative LLD** ^**a**^	6.4	5.7	6.8	6.7	6.5	7.7	6.3	4.9	6.8
**BMI (kg/m** ^**2**^ **)**	27.1	27.0	27.1	27.0	27.5	24.1	27.1	26.5	27.3
**Smoking** ^**b**^	23	23	22	18	21	0	24	26	24
**Depressive feelings**	3.1	2.6	3.4	2.5	2.4	3.2	3.3	2.8	3.4
**Work ability**	8.1	8.1	8.0	8.5	8.5	8.6	7.9	7.7	8.0
**Days with LBP** ^**c**^	53	56	51	28	27	32	60	83	52
**LBP intensity** ^**d**^	3.1	3.1	3.1	2.9	3.1	1.6	3.2	3.2	3.1

LLD = leg length discrepancy, LBP = low back pain; ^a^LLD as a percentage of mean leg length; ^b^The participants were categorized as current smokers if they smoked at least 2 days a week; ^c^Self-reported number of days with LBP during the past year; ^d^Pain intensity during the past week assessed using a 10-cm Visual Analogue Scale.

Customer service workers sit mostly in their work, while meat cutters stand.

**Figure 1:**
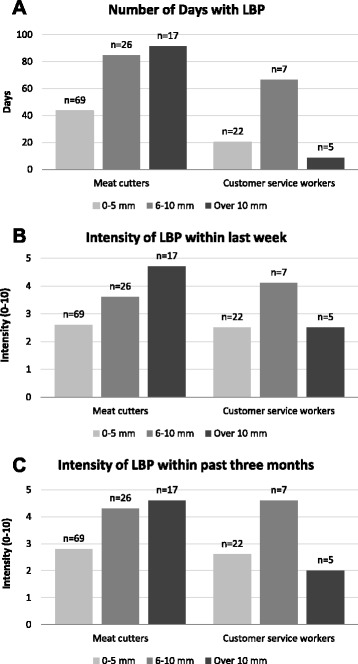
The association of frequency **(A)** and intensity **(B, C)** of low back pain (LBP) with leg-length discrepancy among meat cutters and customer service workers.

In the total study population, LLD of 6 mm or more was associated with higher intensity of LBP during the past week and 3 months in the adjusted analyses (Table 2). As responses were logarithmized in the linear regression analysis, the interpretation of the estimates differs from the analysis using original scaling. The estimated effect of LLD > 5 mm, 0.53, indicates that the mean intensity of pain is approximately 53% greater compared to LLD ≤ 5 mm. Self-reported number of days with LBP was higher with those with LLD 6 mm or more but the probability of the days with LBP was not associated significantly with LLD in the adjusted analyses (Table 3). The sick leaves during past year were slightly longer among those with LLD 11 mm or more, but the differences were not statistically significant (Figure 1).

**Table 2:** **Linear regression analysis on the association of leg-length discrepancy (LLD) of 6 mm or more and other explanatory variables with intensity of low back pain (LBP) during the past week and past 3 months**

	**Intensity of LBP in the past week** ^**a**^		**Intensity of LBP in the past 3 months** ^**a**^	
	**Unadjusted estimate (95% CI)**	**Adjusted** ^**b**^ **estimate (95% CI)**	**Unadjusted estimate (95% CI)**	**Adjusted** ^**b**^ **estimate (95% CI)**
**LLD > 5 mm**				
No	0.00	0.00	0.00	0.00
Yes	0.60 (0.37-0.83)^***^	0.53 (0.32-0.74)^***^	0.54 (0.33-0.76)^***^	0.49 (0.29-0.69)^***^
**Depressive feelings** ^**c**^		0.34 (0.21-0.46)^***^		0.34 (0.22-0.46)^***^
**Gender**				
Male		0.00		0.00
Female		0.23 (−0.02-0.48)		0.31 (0.07-0.54 )^*^
**Smoking**				
No		0.00		0.00
Yes		0.14 (−0.11-0.38)		0.08 (−0.16-0.31)
**Age** ^**d**^		−0.01 (−0.02-0.00)		−0.01 (−0.02-0.01)
**Working position** ^**e**^				
Sitting		0.00		0.00
Standing		0.05 (−0.23-0.33)		0.24 (−0.05-0.48)

^a^Pain intensity assessed using a 10-cm Visual Analogue Scale. Responses are logarithmized, therefore the interpretation of the estimates differs from the analysis using original scaling. Estimated effect of LLD > 5 mm, 0.53, means that mean intensity of pain is approximately 53% greater compared to LLD ≤ 5 mml; ^b^Adjusted for all variables in the table; ^c^Depressive feelings=logarithmized total score from the PHQ-9 questionnaire; ^d^Age in years; ^e^Sitting=customary workers, standing=meat cutters; ^*^p < 0.05, ^***^p < 0.001.

**Table 3:** **Hurdle regression model, with count and hurdle components, on the association of leg-length discrepancy (LLD) greater than 5 mm and other explanatory variables with self-reported number of days with low back pain during the past year**

	**Hurdle**		**Count**	
	**Unadjusted OR(95% CI)**	**Adjusted** ^**a**^ **OR(95% CI)**	**Unadjusted MR(95% CI)**	**Adjusted** ^**a**^ **MR (95% CI)**
**LLD > 5 mm**				
No	1.00	1.00	1.00	1.00
Yes	1.45 (0.67-3.13)	1.35 (0.56-3.20)	2.32 (1.33-4.05)^**^	2.89 (1.63-5.09)^***^
**Depressive feelings**		1.27 (1.07-1.52)^**^		1.09 (1.01-1.17)^*^
**Gender**				
Male		1.00		1.00
Female		0.69 (0.24-1.92)		0.39 (0.19-0.77)
**Smoking**				
No		1.00		1.00
Yes		2.63 (0.80-8.65)		0.53 (0.28-0.97)
**Age**		0.96 (0.90-1.01)		0.99 (0.95-1.02)
**Working position** ^**b**^				
Sitting		1.00		1.00
Standing		2.57 (0.87-7.69)		1.95 (0.93-4.09)

^a^Adjusted for all variables in the table; ^b^Sitting=customary workers, standing=meat cutters; MR=mean ratio and OR=odds ratio; ^*^p < 0.05, ^**^p < 0.01, ^***^p < 0.001.

None of the gender interaction terms in multivariate analyses were statistically significant (p > 0.10). However, as males were significantly taller than females (p < 0.001), we repeated all multivariate analysis using relative LLD as a continuous dependent variable (data not shown). Similar results were obtained, as relative LLD was associated significantly with higher intensity of LBP during the past week (p < 0.001) and 3 months (p < 0.001) and with higher self-reported number of days with LBP (p < 0.01). No statistically significant associations between the probability of the days with LBP or sick leaves and relative LLD were found but the sick leaves were longer among meat cutters (data not shown).

**Figure 2:**
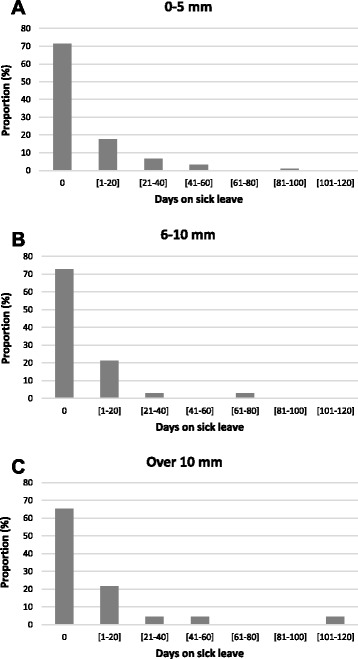
The proportion of workers who reported a given number of days on sick leave according to degree of leg-length discrepancy of 0–5 mm **(A)**, 6–10 mm **(B)** and over 10 mm **(C)**.

There was no significant interaction effect between type of work and LLD regarding intensity or number of days of LBP (p > 0.10). However, both intensity of LBP and number of days of LBP were associated with LLD among meat cutters but not among customer service workers in stratified analysis (Figure 2). Meat cutters who had LLD of 6 mm or more had a significantly higher intensity of LBP during both the past week and past three months compared to those with LLD ≤ 5 mm, while among customer service workers no significant association was found.

## Discussion

In our study LLD (6 mm or more) was associated with a higher likelihood of low back symptoms among meat cutters with a standing and physically more demanding job.

Among chronic LBP patients (n = 1309), almost every fifth had LLD of more than 9 mm compared to only four of 50 voluntary controls who had no history of LBP [4]. Among Finnish soldiers (n = 798) a higher prevalence of LLD was observed among those with LBP than without [5]. In a small-scale study, LBP patients (n = 10) with LLD of 10 mm reported increasing pain while standing for 20–30 minutes, followed by immediate relief upon sitting [6]. On the other hand, among factory workers (n = 594), workers with and without LBP had similar distributions of LLD up to 10 mm [10]. Similarly, no statistically significant difference in LLD was observed between chronic LBP patients (n = 70) and their age- and sex-matched controls [7].

It is possible that the contradictory results in previous studies are due to the differences in study populations and methodology used for measuring LLD. In most of the previous studies [4-6,10] LLD was assessed using erect-posture radiography, while some [7,8] used even the clinical method, which is less reliable and valid than radiographic or ultrasound methods [15,16]. The strength of our study is the validated accurate method to measure LLD [12]. Our laser-based ultrasound measuring method was accurate and easy to use, which makes it suitable for large-scale studies. In our study, the questionnaires were filled before the LLD measurements. The physiotherapists who measured LLD were not aware of workers’ low back symptoms or disease history. All meat cutters were working in the same department and performed similar physically demanding work tasks. Similarly, all customer service workers had identical work description. The characteristics of workers who participated in the study did not differ from those who declined.

In the current study, LLD was associated with low back symptoms after adjustments for confounding factors in the whole occupational sample. However, when occupational groups were analysed separately, the association was observed among meat cutters whereas among customer service workers from the same factory the effect was much smaller and not statistically significant. Yet the distribution of LLD was similar in both groups. Our findings suggest that LLD associated with standing and physically heavier job might render a higher risk for LBP than LLD in sedentary, physically less demanding office work. Among individuals who had LLD in our study, particularly lower extremity loading may have triggered LBP. LLD may cause abnormal biomechanical stress in the pelvis and lumbar spine during walking through asymmetrical loading. The exact mechanism by which LLD causes or augments LBP, however, is not clear. Although our study showed that both absolute and relative LLD were associated similarly with LBP, some anthropometric characteristics might weaken or strengthen the relationship between LBP and LLD.

In the statistical analyses, number of days with LBP had a skewed, highly dispersed distribution with large amount of zeros (zero inflated distribution); therefore Poisson regression could not be used. We considered different models available for zero inflated distribution and chose Hurdle model since some of the workers did not have LBP and hurdle model recognizes the possibility that there can be different factors contributing to probability to have LBP in the first place and to duration of LBP for those who have LBP.

The weakness of our study is the small sample size, especially in customer service workers. Consequently, we were not able to exclusively explore the role of working positions in the estimated associations and further studies should confirm the generality of our conclusions. Our data did neither allow for in-depth investigation of possible gender differences. As most meat cutters were males, a significant gender-specific mechanism behind the association between LLD and LBP, rather than standing or sitting, could potentially explain our findings. However, we believe that this is unlikely, because the associations were independent of leg length. A further limitation is that we did not measure exact time spent sitting or standing in the workplace. However, in the meat factory from which our study sample was drawn, customer service workers mostly sit and meat cutters mostly stand in their work, which result in greatly different overall time spent standing. We relied on self-reports of outcomes and the number of days with LBP during the last 12 months should only be interpreted as a rough proxy for LBP frequency due to long recall period. Finally, cross-sectional study design can only reveal an association but does not explain the interrelationships of different factors. Longitudinal studies are needed to assess whether LLD is a risk factor of LBP. Additional proof for the role of LLD in LBP would be provided by a randomised controlled trial showing a beneficial effect of LLD correction on LBP symptoms.

## Conclusions

Our study found a significant association between LLD of 6 mm or more and low back symptoms. The association was apparent among meat cutters, who stand while working, but not among customer service workers, who mostly sit while working. We hope that future studies would assess whether our results can be replicated with larger sample size and in other populations using a similar reliable assessment method of LLD.
